# Central Pontine Myelinolysis in Pregnancy: A Case of Rare Occurrence

**DOI:** 10.7759/cureus.20281

**Published:** 2021-12-08

**Authors:** Sami Ullah Khan, Muhammad Saqib Saeed, Dawood Misbah, Muhammad Idrees, Abdullah LNU

**Affiliations:** 1 Department of Internal Medicine, Medical Teaching Institution, Ayub Teaching Hospital, Abbottabad, PAK; 2 Department of Neurology, Medical Teaching Institution, Ayub Teaching Hospital, Abbottabad, PAK

**Keywords:** central pontine myelinolysis, gradual onset, osmotic demyelination syndrome, postpartum, pregnancy

## Abstract

Central pontine myelinolysis is a non-inflammatory neurologic deficit and can have a wide array of clinical features, predisposing risk factors as well as different patterns of onset along with a big difference in prognosis ranging from asymptomatic cases to encephalopathy and also mortality. Apart from the common risk factors like hyponatremia and sudden correction of electrolyte imbalances, sometimes, the least prevalent risk factors such as pregnancy seem to link with the central pontine myelinolysis. Mostly its onset is sudden after the inciting factors. However, it is also likely to have cases of central pontine myelinolysis with gradual onset of clinical features. The purpose of the case report is to highlight the link between pregnancy and central pontine myelinolysis. The slow onset of clinical features in pregnancy-linked central pontine myelinolysis can also be considered. The patient in the case report presented with gradual onset clinical features of osmotic demyelination syndrome during the last months of pregnancy and immediately postpartum. All the possible predisposing risk factors for central pontine myelinolysis were ruled out through history, physical examination, and relevant investigations. The case study of the patient hypothesized that: (1) pregnancy should be considered as a risk factor for central pontine myelinolysis in pregnant and postpartum patients presenting with clinical features of the disease, (2) clinical features of central pontine myelinolysis in pregnancy can have a more gradual onset of symptoms compared to other causes of central pontine myelinolysis. Although, this case report signifies a relationship between pregnancy and osmotic demyelination syndrome. However, further studies should be done to develop a causal relationship and preventive measures for the condition.

## Introduction

Central pontine myelinolysis (CPM) is a major subset of a group of disorders called osmotic demyelination syndrome (ODS), in which damage to different parts of the brain occurs, predominantly the white matter pontine tracts [1]. The most common cause is an electrolyte imbalance, particularly hyponatremia, whereas; others include rapid correction of hyponatremia, malnutrition, hepatic failure, alcoholism, malignancies, hemodialysis, and severe burns [2]. Hyponatremia results in decreased serum tonicity, which causes cerebral edema through osmosis, however, the brain has several regulatory mechanisms to tackle this, such as removal of water from brain cells into the cerebrospinal fluid (CSF), efflux of organic osmolytes, and removal of intracellular solutes and water through ion channels to reduce brain swelling. When these corrective mechanisms are coupled with rapid therapeutic correction of hyponatremia, it renders the brain’s capacity to recapture lost osmolytes and hence, leads to dehydration of brain tissue and demyelination of the white matter, especially the astrocytes [1]. CPM has a biphasic presentation, initially with seizures secondary to hyponatremia which resolve with correction of the imbalance, and then with a wide range of symptoms depending on the area involved which may include dysarthria, dysphagia, quadriparesis, pseudobulbar palsy, and pseudocoma referred to as "locked-in syndrome". The chief diagnostic tool remains to be magnetic resonance imaging (MRI) [3]. Interestingly, both the causes and presentations of CPM were atypical in many of the case reports. For instance, hyperglycemia was the cause in one of the cases, and a patient presented with neuropsychiatric symptoms in another [4,5]. Nevertheless, the prognosis of ODS including CPM remains good with more than 50% recovery rate even in those with severe neurological symptoms and tends to keep getting better with each passing decade owing to early detection of ODS and the availability of better diagnostic tools [6]. In the past, young postpartum women have presented with extra pontine myelinolysis; however, to the best of our knowledge, none have presented with central pontine myelinolysis [7]. Here, we present a case of a 25-year-old post-natal woman who presented with loss of consciousness, quadriparesis, and progressive dysarthria.

## Case presentation

**Figure 1:**
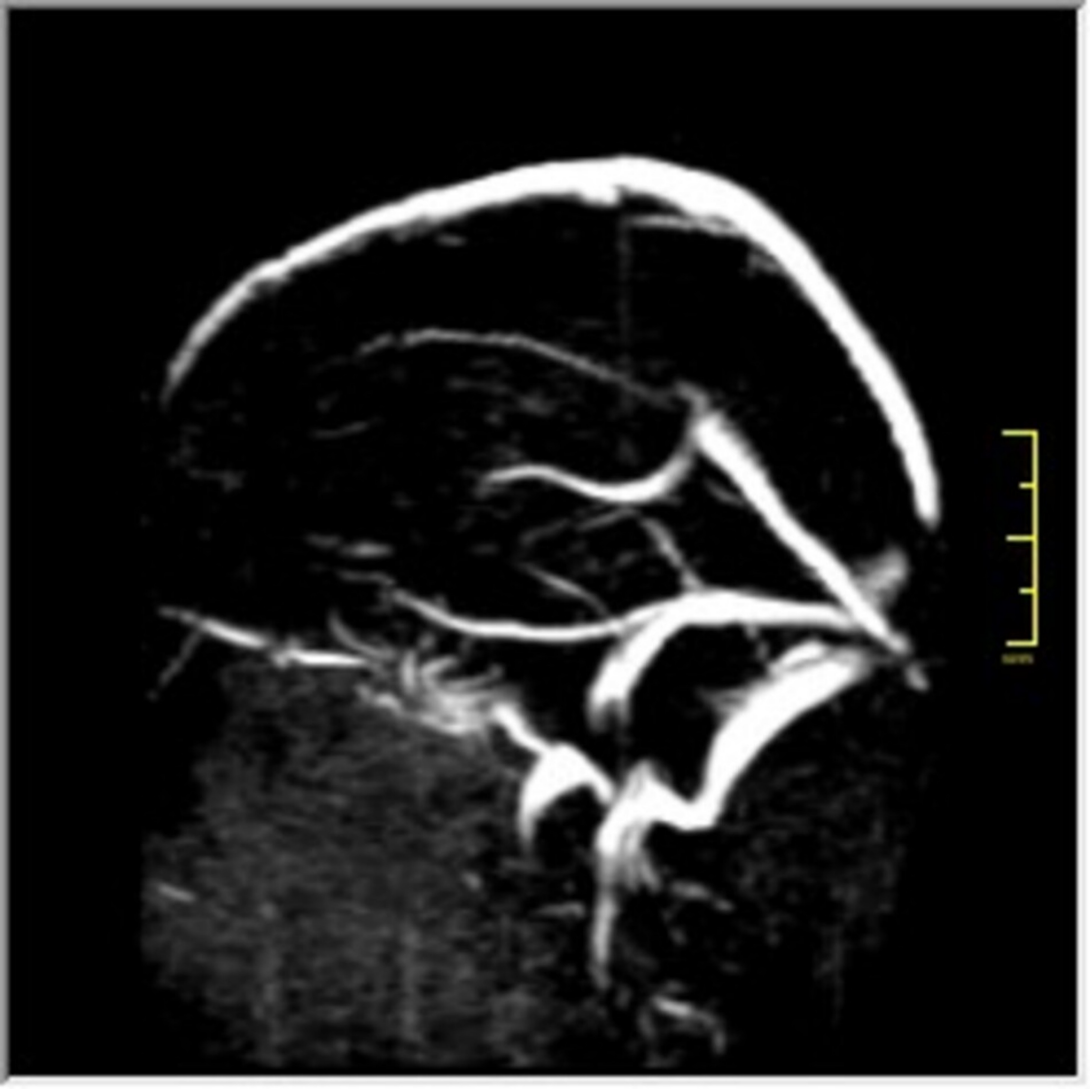
MRV brain. MRV: magnetic resonance venogram.

**Figure 2:**
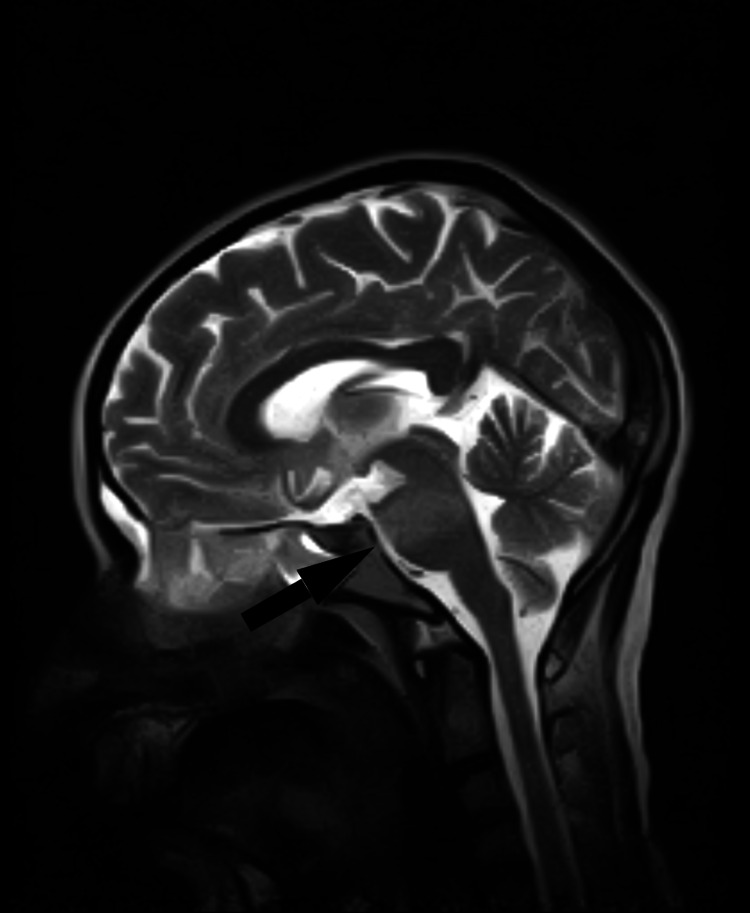
MRI brain, sagittal, T2-weighted image shows a subtle abnormal hyperintense signal in the region of pons (arrow). MRI: magnetic resonance imaging.

A 25-year-old, 12 days post-natal female patient presented to accidents and emergency department with loss of consciousness. On inquiry, her husband said that she was fine a month ago when she started developing dysarthria which was gradual in onset, progressive with no aggravating and relieving factors. The patient also started experiencing gradual and progressive bilateral upper and lower limbs weakness. These symptoms were followed by diplopia and progressive difficulty in swallowing. Obstetric history revealed no prenatal care. There was no significant past medical and neuropsychiatric history. On general physical examination, BP was 120/80 and pulse was 82/min with a regular rhythm. On neurological examination, she had poor eye-opening and vocalization with a Glasgow Coma Scale (GCS) of 5/15. Corneal reflexes were intact. The eyes of the patient did not turn with the head-turning (positive Doll’s eye reflex). On motor examination, the patient had decreased purposeful hand movement with diminished tone and reflexes in all four limbs. All baseline investigations, including complete blood count, renal function tests, liver function tests, serum electrolytes, and CSF analysis, were normal. Magnetic resonance venogram (MRV) brain (Figure 1) had no evidence of dural sinus thrombosis. Magnetic resonance imaging (MRI) brain (Figure 2) showed subtle abnormal hyperintense signals on T2-weighted image (T2WI) and fluid-attenuated inversion recovery (FLAIR), seen in the region of pons sparing the peripheral parts suggestive of central pontine myelinolysis. The treatment plan included supportive treatment such as nursing care, airway care, chest physiotherapy as well as vitals monitoring of the patient. The patient’s condition was static during the hospital stay, although she had mild improvement in her response to pain. Her family was counseled regarding the prognosis of the disease and palliative care. Finally, she was discharged, and a follow-up appointment was scheduled.

## Discussion

Osmotic demyelination syndrome encompasses a wide array of causes, spectrum, and risk factors, along with clinical and radiological pictures. The most common reason for osmotic demyelination syndrome is hyponatremia along with rapid correction of electrolyte imbalance; which came out to be the second most important risk factor [6]. Apart from that, there are some other less important risk factors like pregnancy for which cases have been reported in the past. The patient discussed was postpartum and had an onset of symptoms one month prior to delivery. Most of the time the onset is sudden, but there are cases with gradual onset of symptoms [8]. The clinical features of the patient mentioned in the study gradually progressed over two and a half months.

## Conclusions

The presentation and risk factors for osmotic demyelination syndrome can vary significantly from patient to patient. Keeping this in view, pregnancy should be considered as a risk factor for osmotic demyelination syndrome in any pregnant and postpartum patient presenting with clinical features suggestive of the disease. Also, clinical features of osmotic demyelination syndrome can have a more gradual onset of symptoms as compared to other causes of central pontine myelinolysis. Therefore, timely diagnosis and treatment are necessary.
